# It’s time for a practical method quantifying vascular calcification

**DOI:** 10.1186/1479-5876-12-172

**Published:** 2014-06-17

**Authors:** Giuseppe Vezzoli, Alessandro Rubinacci, Monica Lazzaroni, Laura Soldati

**Affiliations:** 1 Nephrology and Dialysis Unit, San Raffaele Scientific Institute, Via Olgettina 60, 20132 Milan, Italy; 2 Bone Metabolism Unit, San Raffaele Scientific Institute, Milan, Italy; 3 Clinical Pathology, C. Besta Neurological Institute, Milan, Italy; 4 Department of Health Sciences, Università degli Studi of Milan, Milan, Italy

## 

Cardiovascular disease is the main cause of morbidity and mortality in patients with chronic kidney disease (CKD). They present a 5-fold increase in cardiovascular risk at the end-stage of the CKD [1]. Vascular damage in CKD patients is characterized by calcification of the tunica media in the artery wall [2]. Therefore, artery calcification is an independent risk factor for mortality in CKD and its assessment is relevant for the clinical practice. Artery calcification may be estimated with radiographic or echographic methods exploring large arteries or coronary vessels. Multislice computed tomography (MSCT) or electron beam computed tomography (EBCT) are today considered as the gold-standard to quantify calcified deposits at the coronary arteries and aorta. The CT image analysis yields a calcification score that has been correlated with the severity of cardiovascular disease [3]. However, CT methods are expensive, take a relative long time and the radiation dose is not irrelevant. Since these limits, plain X-ray methods are often preferred to CT to evaluate vascular calcification in the clinical practice. The lateral plain radiography of the abdomen allows quantifying lumbar aorta calcification with a score standardized by Kauppila and coll in 1997 [4]. This semiquantitative method estimates calcified deposits at the posterior and anterior wall of the lumbar aorta in front of each vertebral body from L1 to L4, separately. The sum of the calcification score at each segment of the aorta wall yields a score varing from 0 to 24. This method is cheap and practical; however, the computed score depends from the operator ability in identifying deposits and their definition in the radiographic image. Despite these limits, this method has been clinically validated in studies showing its capability to predict cardiovascular risk and its correlation with the score yielded by the EBCT [5,6]. In addition to radiographic methods, ultrasonography has been used to explore artery calcification although a standardized score to quantify calcification has not been developed. Ultrasonographic methods can also measure cardiac calcification and the intima-media thickness of the carotid artery. This method is safe and feasible, but is also operator dependent [7].

**Figure 1:**
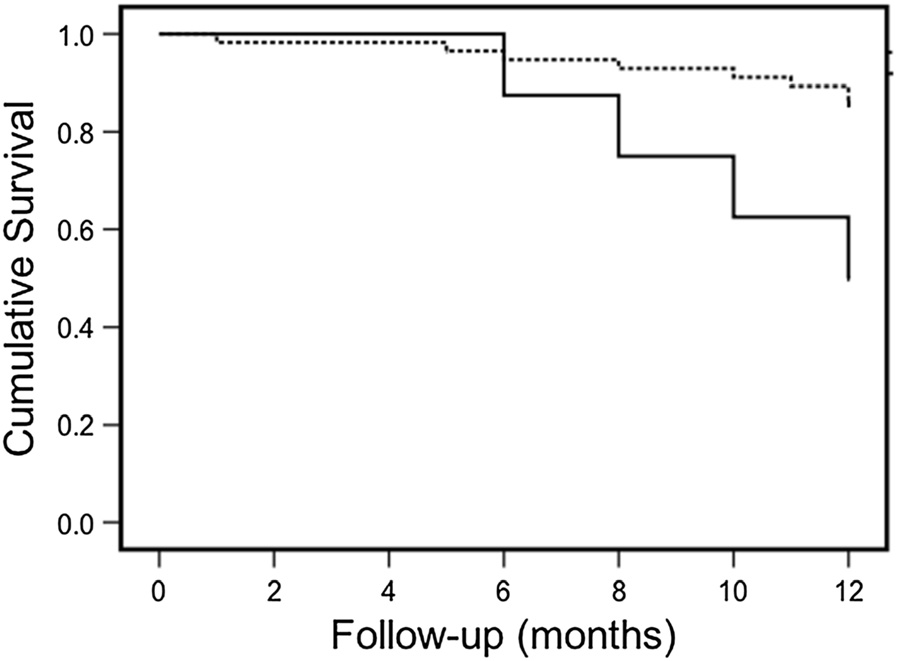
**Kaplan-Meier curve for the first cardiovascular event in CKD patients enrolled for the CREMA study.** Five cardiovascular events occurred in patients with DXA calcification score 5-8 (35% of 14 patients; full line in the graph), while 9 events occurred in patients with score 0-4 (11.1% of 78 patients, p=0.02; dotted line).

Alternative to these methods dual-energy X-ray absorptiomentry (DXA) has been proposed to detect calcification by lateral analysis of the lumbar aorta. Aorta deposits may be estimated with a 24-point or 8-point semiquantitative scale that quantifies calcified deposits in the posterior and anterior wall of the lumbar aorta in front of vertebral bodies from L1 to L4. This calcification score was positively correlated with that yielded by plain X-ray in general population [7] and by CT in dialysis patients [8]. DXA score was a predictor of incident myocardial infarction and stroke in a population of older women [9]. An Italian study (CREMA study) observed the association of DXA score with cardiovascular events in patients with CKD at different stages during a two-year follow-up. The study included 92 patients: 27 undergoing dialysis, 38 with CKD at stage 3, 21 with CKD at stage 4 and 6 with CKD at stage 5. DXA aorta calcification score was determined at patient enrollment and expressed with the 8-point scale. At the end of the first year of follow-up 14 cardiovascular events occurred, including four deaths and ten other acute events. One patient died for non-cardiovascular reason. Nine events occurred in patients having calcification score between 0 and 4 (11.1% of 78 patients), five events in patients having score between 5 and 8 (35.7% of 14 patients, p = 0.02). Kaplan-Meier curve of the first cardiovascular event showed that patients with score 5–8 had a larger number of cardiovascular events during the year of follow-up compared with patients with score 0–4 (p = 0.02, Figure 1). Cox multivariate regression analysis showed that patients with calcification score of 5–8 had a 13-time higher cardiovascular risk adjusted for the other confounding factors, relative to the patients with score 0–4 (OR = 13, 95% confidence interval 1.4-118, p = 0.023). DXA calcification score was correlated with the Kauppila's score, but showed a greater association with cardiovascular events compared with the Kauppila's score. These findings support the clinical value of the DXA method to predict cardiovascular risk in CKD patients. DXA method is cheap, needs a shorter time and exposes patients to a very low x-ray dose [10]. Despite these practical advantages, the obtained DXA score is semiquantitative and operator dependent. These aspects limit the large application of DXA scanning of the abdominal aorta to the clinical practice. However, current perfomance of the DXA evaluation of the aorta wall could be largely improved by developing automated algorithms for the computation of the abdominal aorta calcification score on a continuous scale. This potential evolution to a quantitative method might overcome current limits of the DXA assessment and is theoretical feasible given the high resolution achieved by the most recent generation of DXA digital detectors. This could be a good challenge for the companies producing DXA equipments.
